# Increasing Cyber Defense in the Music Education Sector Using Blockchain Zero-Knowledge Proof Identification

**DOI:** 10.1155/2022/9922167

**Published:** 2022-06-28

**Authors:** Ying Zhang

**Affiliations:** Zhengzhou Preschool Education College, Zhengzhou, Henan 450000, China

## Abstract

Music creation and its promotion are encouraged both in music education and through activities organized in the context of artistic creation as part of the education in question. Although copyright registration is the primary way authors protect their rights, this is not feasible in most cases, as the processes take a long time to complete and incur high costs. We utilize modern innovative technologies and their developments in copyright protection matters to increase security and trust in music education. In particular, an advanced model of ensuring the methods and innovation produced in music education processes is proposed, using blockchain technology and smart contracts. But given that, even in an advanced system like the proposed one, authentication evidence can be easily intercepted, this work proposes a single and robust identification scheme based on an innovative zero-knowledge proof (ZNP) system, which allows one side of communication to convince the other of its validity.

## 1. Introduction

In recent years, cloud computing has been widely used in many areas of everyday life, mainly for data storage [1, 2]. This raises questions about the reliability and how to manage the data in question. The multitude of these services targets widespread attacks by third parties. These attacks find fertile ground as they exploit security vulnerabilities, resulting in data leaks [3]. The result is that both the security and the privacy of the data stored in cloud services are questioned [4, 5]. In addition, users' data is often used for exploitation purposes or given to third parties such as advertising companies. Another deterrent is that data providers usually store that information without encryption, making user data easily accessible [6].

An attractive solution that can give another approach to the issue is blockchain, which proposes a decentralized and highly secure solution for data storage [7]. Similarly, the blockchain can be used as an intermediary whose primary function is to maintain and validate actions within the chain. In general, blockchain implements a distributed global platform that runs smart contracts [8], utilizes proven technologies, and has an architecture that allows various additional functions to be implemented simply and transparently [9]. It enables the creation of different security levels and licenses only certified users to access specific services or resources. Due to the encryption [10, 11] of transactions and its operating environment, it is ideal for environments that require reliability without the mediation of third-party trusted entities, as it can achieve complete confidentiality of transactions and selective access between participants only to licensed information. This achieves the confidence of the participants in the sharing of information, combined with all the benefits of blockchain [12, 13].

This function is advanced further by using blockchain-provided smart contracts, which allow access to information under precise, strictly specified, and preagreed-upon conditions [8, 14]. When unavoidable circumstances are met, these contracts will close deals. It is simply a protocol designed to digitally facilitate, verify, or enforce the negotiation or execution. These contracts enable the performance of trustworthy transactions without the involvement of third parties, with the transactions being secure, monitored, and irreversible [15, 16]. They seek to protect the scope of contract law while also reducing the additional processing expenses involved with the award and implementation of intermediary contracts [17]. Blockchain implementation is based on the Byzantine Fault Tolerance (BFT) consent algorithm [18, 19], which means that its command service must be jointly controlled by network members. Using the BFT algorithm, the standard guarantees coverage or the ability to reach consensus, even if there are rival nodes (malicious) or if the nodes are offline [20].

In this paper, we present an enhanced model that uses blockchain technology and smart contracts to guarantee the approaches and innovation achieved in music education. Given that, even in a progressive system like the one that is being proposed, the proposed approach is that the authentication items can be easily copied, guessed, or revealed by automated methods and technical means, the main contribution of this work is to offer a single and robust identification scheme, which is based on an innovative ZNP system [12], which enables one side of communication to convince the other side of its validity.

The following is the structure of the paper. In the following section, an overview of the several appropriate methods that have been identified in the relevant literature is presented. In Section 3, we will discuss the ZNP protocol that has been delivered. In Section 4, the scenarios and results prove that ZNP and complexity exist. In the final part of the report, Section 5, a summary of the findings and a list of possible following study directions are presented.

## 2. Related Literature

The literature on blockchain technologies, smart contracts, and zero-proof knowledge is becoming more and more frequent since these innovative technologies are gaining confidence in the community [21].

Hu et al. [14] gave a detailed study of smart contracts, emphasizing current applications and the problems they confront. They introduced the idea of a blockchain-based smart contract, a digital software meant to enable the settlement or contract terms immediately among users when specific circumstances are satisfied. With the improvement in blockchain technology, smart contracts are being utilized to fulfill a wide variety of objectives, from self-maintained accounts on public blockchains to automating corporate collaboration on blockchain systems.

On the other hand, Wang et al. [8] provided a systematic and extensive assessment of blockchain-enabled intelligent contracts to motivate more study in this developing research field. Following the introduction of the operational mechanism and mainstream platforms for blockchain-enabled smart contracts, they proposed a scientific framework for smart contracts based on a groundbreaking six-layer design, which was accepted by the scientific community. Second, the technological and legal difficulties, as well as current research advances, were detailed. Thirdly, they discussed some representative application cases. They concluded by debating the future development patterns for smart contracts.

Yang and Li [12], employing smart contracts and zero-knowledge proof methods to create identity unlikability, have successfully avoided the disclosure of attribute ownership in the present claim identification model on the blockchain. Aside from that, they created a system prototype known as BZDIMS, which features a challenge-response protocol that allows users to reveal their ownership of characteristics to service providers, thereby maintaining the privacy of their activities. Performance and security analyses demonstrated that their system provided good attribute privacy security and a broader application breadth than the previous paradigm.

Sankar et al. [17] examined and compared the viability and efficiency of blockchain consensus algorithms. The consensus protocol is at the heart of blockchain technology. Academics are eager to design a well-optimized Byzantine fault-tolerant consensus system in light of the advent of new possibilities in blockchain technology. Exciting options include developing a worldwide consensus protocol or creating a cross-platform plug-and-play software application to support a variety of consensus mechanisms. Incorporating the principles of quorum slices and federated Byzantine Fault Tolerance, the Stellar Consensus System is a global consensus protocol designed to be fault-tolerant and claims to be Byzantine Fault Tolerance. Additionally, the hyperledger is an open-source project led by the Linux Foundation that focuses on realizing the notion of realistic Byzantine Fault Tolerance and providing a framework for the plug-and-play deployment of many different consensus protocols and chain applications.

Finally, Buchman [18] developed Tendermint, a novel protocol for organizing events in a dispersed network under adversarial circumstances, as part of his examination of Byzantine Fault Tolerance. Known more frequently as unanimous agreement or atomic broadcast, the problem has gained significant attention in recent years because of the widespread growth of digital currencies such as Bitcoin and Ethereum, which effectively remedy the issue in public settings without the intervention of a central authority. Their concept modernized previous academic work in the field by providing a safe consensus mechanism with accountability requirements and functionality for creating arbitrary applications atop the consensus. Their idea is a high-performance blockchain, capable of processing several events per second over dozens of nodes scattered across the world, with a latency of less than one second and performance deteriorating very slightly in the face of hostile assaults.

## 3. Proposed ZNP Protocol

Entering a service electronically involves different authentication methods. It often requires repetition of the same information or distinct numbers and codes, which can be easily intercepted or revealed [22]. The service provider usually keeps a summary of each user's password. Each time the user wants to connect to the service, the password is given in the summary function, and the result is compared to the saved one [23]. This protocol may not allow the password to be saved in its original form, but the server temporarily learns it [24, 25]. This process could be replaced with a ZNP indicating that each customer owns the password [12].

Although it has offered us many benefits, including openness, immutability, and decentralization, blockchain technology may not provide the necessary level of anonymity for certain types of transactions. However, integrating blockchain technology with ZNP has the potential to deliver to customers a potent combination of immutability and security. A ZNP is a sort of cryptography that allows one person (the prover) to demonstrate to another party (the verifier) that certain information is accurate without giving any extra information. When it comes to messaging applications, end-to-end encryption has been a significant factor in developing private message transmission. On the other hand, traditional messaging applications demand that users verify their identities on a central server. Individuals can demonstrate their identity using ZNPs without divulging any more personal information.

In the proposed ZNP, each calculation is performed by exchanging messages between an entity called prover (*P*) and an entity called verifier (*V*). Typically, *P* wants to convince *V* that a proposition is true (witness). *P* and *V* are probabilistic Turing machines, where *P* has unlimited computing power while *V* is limited to probabilistic calculations of polynomial complexity [26].

Zero knowledge is realized, given that *V* learns nothing more than the fact that *P*'s claim is valid [27]. A key role in proving that an interactive system has the property of zero knowledge is played by the simulator (*S*), which simulates *P* but does not have access to the witness. His contribution is as follows [28]: *V* interacts with *S*. At some point, *V* will put *S* in the “difficult position” of not being able to answer a question as he does not have access to the witness. In this case, we return the *V* tape to a state before rewinding and running the protocol from that point on. If *V* (with continuous rewinds) finally accepts *S*'s proof, the protocol holds the status of zero knowledge, as *V* cannot distinguish a *P* who knows the witness and an *S* who pretends. *V* cannot export any additional information from the protocol (since, in the second case, there is no information to ship) [29, 30].

Let an NP language *L* and *M* be a polynomial Turing machine such that [31, 32]

(1) $$\begin{array}{c} x\in L\Leftrightarrow \exists w\in {\left\{0,1\right\}}^{p\left(\left|x\right|\right)}:M\left(x,w\right)=1, \end{array}$$

where *p* is a polynomial. One proof of zero knowledge for *L* is two possible Turing Polynomial Time (TPT) machines *P* and *V* for which the following three properties apply [33]:

(1) Completeness: if *x* ∈ *L* and *w* are a witness to this, that is,
  (2) $$\begin{array}{c} M\left(x,w\right)=1, \end{array}$$
then
  (3) $$\begin{array}{c} \mathrm{Pr}\left[{\text{out}}_{V}<P\left(x,w\right),V\left(x\right)>\left(x\right)\right]=1\geq \frac{2}{3}, \end{array}$$
where
  (a) *P*(*x*, *w*), *V*(*x*) is the interaction between *P* and *V* with standard (public input) *x* and private input of *P* at *w*.
  (b) out_*𝒱*_ is the output *V* at the end of the protocol.
(2) Correctness: if *x* ∉ *L*, then
  (4) $$\begin{array}{c} \forall \left(P^{*},w\right)\mathrm{Pr}\left[{\text{out}}_{V}<P^{*}\left(x,w\right),V\left(x\right)>\left(x\right)\right]=1\leq \frac{2}{3}.OP^{*}, \end{array}$$
where *P*^*∗*^ does not need to be TPT.
(3) Validity: *V* does not accept false statements (even if *P* tries to trick him).

The proposed model appears to be related to the NP complexity class in the above definition [34–37].

## 4. Evidence of ZNP and Complexity

To prove the proposed ZNP methodology, we will use three different examples which show its power as a computational and cryptographic model which can respond to the proposed implementation [15, 38].

### 4.1. Graph Isomorphism

**Figure 1:**
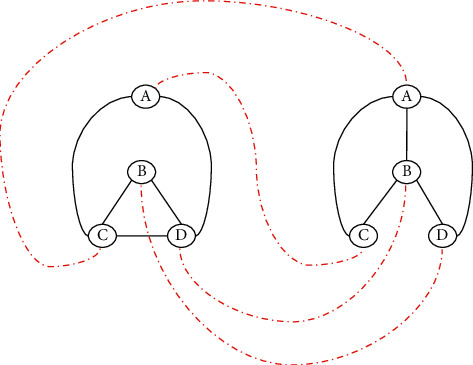
Two isomorphic graphs where the mapping from ABCD to CDAB corresponds to the first graph to the second.

The first example concerns the isomorphism of graphs. Specifically, two isomorphic graphs where the mapping from ABCD to CDAB corresponds to the first graph to the second, as shown in Figure 1.

Two graphs, *G*1 and *G*2, are said to be isomorphic if they have the same number of vertices. There is a shift, that is, function 1–1 and on, between their nodes such that two nodes of one are connected by acne if and only if the corresponding nodes of the other are connected by acne. Equivalently, there is a renaming of the nodes of a graph such that the charts are identical. The problem of graph isomorphism belongs to the NP class, but it is not known whether it is NP-complete or not [39]. Assume that both *P* and *V* know the graphs *G*1 and *G*2; that is, the latter is a common input of the protocol. In addition, *P* knows the isomorphism between them *ϕ*: *G*1 ⟶ *G*2 (private input of *V* or the witness mentioned above). Using a zero-knowledge protocol, he can prove that he knows the isomorphism without revealing it [40]:

(1) *P* randomly selects one of *G*1, *G*2, and *Gi*. By some permutation *ψ* of the vertices of *Gi*, *P* produces the graph *H* = *ψ*(*Gi*), which is isomorphic with *Gi*. Because *P* knows the isomorphism *ψ* between *H* and *Gi*, he also knows the isomorphism *ψϕ*˙ between *H* and *G*3 − *i*. Anyone else has as much difficulty finding an isomorphism between *H* and *G*1 or between *H* and *G*2 as finding an isomorphism between the initials *G*1 and *G*2.
(2) *P* binds to *ψ*, sending *H* to *V*.
(3) *V* randomly selects a graph from *G*1, *G*2, and *Gj* and sends his selection as a challenge to *P*, asking him to prove that *H* and *Gj* are isomorphic. That is, he asks for a permutation of *Gj* to produce *H*.
(4) *P* responds by doing the following:
   
$\left(\hat{I}\pm I^{\prime }\right)\text{if }G_{i}=G_{j}$, send to *V* the permutation *ψ*.
   
$\left({\hat{I}}^{2}I^{\prime }\right)\text{if }G_{i}\neq G_{j}$, then we have the following:
  (i) If *G*1 and *G*2 are isomorphic (then ∃*ρ* : *G*_*i*_=*ρ*(*G*_*j*_)), send to *V* the permutation *ψρ*
  (ii) If *G*1 and *G*2 are not isomorphic (i.e., *P* is not honest), then it cannot find a suitable permutation and sends any random permutation
(5) If *V* receives a correct permutation, he continues (repeat steps 1–5); otherwise, he stops rejecting (i.e., he considers that the graphs are not isomorphic).

If *V* has not rejected after *k* repetitions of steps 1–5, he accepts (considers the graphs isomorphic). The above protocol fulfills the properties of the zero knowledge mentioned above. First, it is complete because if there is an isomorphism between *G*1 and *G*2, then *P* will convince *V* with a probability of 1 (*V* never rejects) [27, 41].

Regarding correctness, if there is no isomorphism, then *P* has a 1/2 chance at each step to deceive *V* (this will only happen if *Gi* = *Gj*). After *k* repetitions, this probability becomes 1/(2^*k*^).

*V* does not get any additional information regarding the isomorphism between *G*1 and *G*2 regarding the zero knowledge. When interacting with *S*, his first step will be the same as *P*; that is, he will make a new random graph isomorphic to one of *G*1 and *G*2 each time. The probability of choosing either *G*1 or *G*2 is precisely 1/2. So, at this stage, *V* cannot separate them. Thus, the likelihood of cheating in *k* repetitions remains 1/(2^*k*^). So, the expected execution time is polynomial as it results from the relation [15, 39, 42]:

(5) $$\begin{array}{c} T_{V}\frac{\sum_{k=1}^{\infty }1}{\left(2^{k}\right)}=T_{V}, \end{array}$$

where *T*_*𝒱*_ is the execution time of *V*, which is polynomial.

### 4.2. 3-Coloring

A zero-knowledge protocol for an NP-complete problem would mean that all NP problems have zero-knowledge protocols [12, 26, 32, 39]. In the NP-complete problem of 3-Coloring, *P* knows a coloring *c* for a graph *G* = (*V*, *E*) such that [43]

(6) $$\begin{array}{c} c:V⟶\left\{1,2,3\right\}\text{and }c\left(v_{1}\right)\neq c\left(v_{2}\right)\Leftrightarrow \left(v_{1},v_{2}\right)\in E. \end{array}$$

He wants to prove this knowledge to *V* without revealing *c*:

1. *P* selects a random permutation *π* of {1, 2, 3}. From this, an alternative 3-*π* · *c* of *G* then uses a commitment scheme for *π*.*c*, that is, calculates values commit((*π*.*c*)(*v*_*i*_), *r*_*i*_), ∀*v*_*i*_ ∈ *V* and sends them to *V*.
2. *V* selects a random edge (*v*_*i*_, *v*_*j*_) ∈ *E* and sends it to *P*.
3. *P* releases the values *π* · *c*(*v*_*i*_), *π* · *c*(*v*_*j*_) and sends them to *V*.
4. *V* checks if *π* · *c*(*v*_*i*_) ≠ *π* · *c*(*v*_*j*_).

It is evident that the above protocol is complete. Regarding the correctness, we observe that if *P* does not have a valid 3-color, then *V* will choose an edge with the same peak colors with probability 1/|*E*|. By repeating the protocol, we can make the probability that prover 1 − 1/|*E*| cheats him extremely small. About zero knowledge, even S does not have a valid coloring. If *V* chooses an edge with the same peak colors, then it rewinds to a previous state, and *S* selects a new random permutation that it uses in the new execution. It can be shown that the protocol with *S* does not have an expected execution time of a different order of magnitude than with *P* and *V* does not understand the difference. So, the protocol has the property of zero knowledge [15, 42].

### 4.3. Noninteractive Proof of Zero Knowledge

To make a noninteractive proof, we use a hash function [26, 39]:

(7) $$\begin{array}{c} H:{\left\{0,1\right\}}^{*}⟶Z_{q}, \end{array}$$

such that the discussion

(8) $$\begin{array}{c} \left(y,c,s\right)=g^{t},H\left(g^{t}\right),t+H\left(g^{t}\right)w\mathrm{mod}q. \end{array}$$

**Figure 2:**
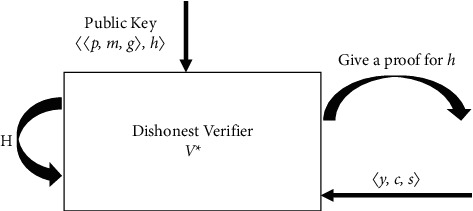
In the random oracle model, the simulation of dishonest verifier *V*^*∗*^ is performed.

Assume that *H* is a random oracle controlled by the simulator to demonstrate that ZNP holds. In the random oracle model, a nonhonest verifier *V* can ask questions of the random oracle and receive answers. In this case, *c* is forced to be selected after *y*, which is directly dependent on the characteristics of the hash function [12, 13].  Figure 2 shows how *V*^*∗*^ interacts with *H*.

When the verifier asks for the proof of *h*=*g*^*w*^, the simulator randomly selects *c* and *s* to compute *y*=*g*^*s*^*h*^−*c*^. Set (*y*, *c*) in History and return 〈*y*, *c*, *s*〉. The nonhonest verifier cannot separate an honest prover from an emulator unless (*y*, *c*′)∈ History with *c* ≠ *c*′. Then, *V*^*∗*^ achieves with probability (1/*m*)*q*_*H*_, where *q*_*H*_ is the number of questions in the random oracle. Then, we want to produce two discussions that end in acceptance with the same *y* but with different challenge values. Using these two discussions, we can extract a witness. Note that *c*=*H*(*y*). If a dishonest prover *P*^*∗*^ asks a unique question in the random oracle before producing 〈*y*, *c*, *s*〉, the resolution is the same as the interactive protocol. Problems arise when *P*^*∗*^ asks more than one question [31, 38, 39].

Assume that, in the first round, *P*^*∗*^ asks *q*_*H*_ questions before ending the discussion. The knowledge exporter then returns *P*^*∗*^ to a previous step, with no guarantee that *P*^*∗*^ will request *q*_*H*_ questions again. When *P*^*∗*^ finishes, it will return *y*′, *c*′, *s*′ with *c*′=*H*(*y*′) and possibly *y* ≠ *y*′. This limits our capacity to compel a witness to testify, so we should modify the probability of having two acceptance discussions with the same *y*.

Assume that, after asking *q*_*H*_ questions, *P*^*∗*^ selects a question he asked and uses the corresponding answer he got for it from the random oracle at his exit. Let Prob[*A*]=*α* be the probability that the discussion will end in acceptance. Let Prob[*Q*_*i*_]=*β*_*i*_ be the probability that the dishonest prover uses the *i*-th answer *c*_*i*_, in which 1 ≤ *i* ≤ *q*_*H*_. We define Prob[*A*∩*Q*_*i*_]=*α*_*i*_. Respectively, for the repetition of the experiment, we write *A*′, *Q*_*j*_′, *c*_*i*_′. Then, it is valid [15, 29, 30, 42]:

(9) $$\begin{array}{c} \sum_{i=1}^{q_{H}}\alpha_{i}=\alpha \text{ and}\sum_{i=1}^{q_{H}}\beta_{i}=1. \end{array}$$

We define Prob[*E*] as the probability of extracting a witness from *P*^*∗*^, and we have

(10) $$\begin{array}{c} \text{Prob}\left[E\right]=\text{Prob}\left[A\cap A^{\prime }\cap \left(i=j\right)\cap \left(c_{i}\neq c_{j}^{\prime }\right)\right]. \end{array}$$

Similarly, we have

(11) $$\begin{array}{c} \text{Prob}\left[E\right]\geq \text{Prob}\left[A\cap A^{\prime }\cap \left(i=j\right)\right]-\text{Prob}\left[\left(c_{i}=c_{j}^{\prime }\right)\right]. \end{array}$$

So,

(12) $$\begin{array}{c} \text{Prob}\left[E\right]\geq \text{Prob}\left[A\cap^{}A^{\text{'}}\right]-\frac{1}{q}=\sum_{i=1}^{q_{H}}\text{Prob}\left[A\cap^{}Q_{i}\cap^{}A^{\text{'}}\cap^{}Q_{i}^{\text{'}}\right]-\frac{1}{q}\\ =\sum_{i=1}^{q_{H}}\text{Prob}\left[A_{i}\cap^{}A_{i}^{\text{'}}\right]-\frac{1}{q}. \end{array}$$

From the definition of exporter in our calculations, we know that

(13) $$\begin{array}{c} \text{Prob}\left[A_{i}\cap^{}A_{i}^{\prime }\right]\geq \frac{\text{Prob}{\left[A_{i}\right]}^{2}}{4}=\frac{\alpha_{i}^{2}}{4}. \end{array}$$

The total probability is calculated as follows:

(14) $$\begin{array}{c} \text{Prob}\left[E\right]\geq \sum_{i=1}^{q_{H}}\text{Prob}\left[A_{i}\cap^{}A_{i}^{\text{'}}\right]-\frac{1}{q}=\frac{1}{4}\sum_{i=1}^{q_{H}}\alpha_{i}^{2}-\frac{1}{q}. \end{array}$$

From the statistics, we know that

(15) $$\begin{array}{c} \frac{\sum \left(\alpha_{i}^{2}\right)}{q_{H}}\geq {\left(\frac{\alpha }{q_{H}}\right)}^{2}. \end{array}$$

And so,

(16) $$\begin{array}{c} \sum \left(\alpha_{i}^{2}\right)\geq \frac{\alpha }{q_{H}}. \end{array}$$

And for any real *α*_*i*_, they have an average:

(17) $$\begin{array}{c} \frac{\alpha }{q_{H}}. \end{array}$$

As a result, we infer that we have a good chance of extracting a witness, given a persuasive prover:

(18) $$\begin{array}{c} \frac{\alpha^{2}}{4q_{H}}-\frac{1}{q}. \end{array}$$

If it is necessary for the person who is proving a statement to possess certain confidential knowledge, then the person who is verifying the statement will not be able to prove the statement to anyone else unless they also possess the confidential information. The assertion that the prover possesses such information must be included in the statement that is being proven, but the knowledge itself cannot be included in the assertion, nor can it be transmitted with it. If this were not the case, the statement could not be proven using the zero-knowledge proof method since it would present the verifier with more information about the statement by the time the protocol was completed. A proof of knowledge is considered to be in the particular situation of zero knowledge when the assertion consists of nothing more than the fact that the prover is in possession of the confidential information.

As proved, concerning zero knowledge, even a node that does not hold a piece of valid information will rewind to a previous state and choose a new random permutation employed in the new execution. This is because zero knowledge prevents a node from storing any information at all. It is possible to demonstrate that the protocol with random nodes does not have an expected execution time of a different order of magnitude. Still, this protocol does not comprehend the distinction. Therefore, the protocol possesses the quality of not revealing any information.

## 5. Conclusions

This work proposed an innovative ZNP system [12] to ensure the methods and innovation produced in music education processes, using blockchain [9, 42] technology and smart contracts [14, 44]. The motivation for the development of this protocol is that, in the “conventional” authentication protocols [39, 45], at the end of their execution, the member who verifies the identity of his peer has messages and secrets that he can use for impersonation [37, 46]. Contrary to the proposed standard, the secret used to prove a member's identity depends on a specific time, so that, at another time, it is useless. In other words, the musical educational processes and the participants may know a secret, but without revealing any information about this secret. Three different examples were used to demonstrate the capability of the template as both a computing and a cryptographic model, capable of responding to the suggested implementation and ensuring the authentication processes of blockchain technology.

Even though it may be possible to achieve a level of protection in musical educational processes that are practically acceptable, it is evident that a significant amount of research work is still required because the requirements are high and are continually increasing [47]. The sheer number of potential solutions and the associated expenses illustrate how challenging it is to ensure the safety of a comparable system in a safe setting. It is acceptable to conclude that, to secure it, specialized methods of issuing identities to the blockchain nodes, scattering the nodes, instant data copying, and an access mechanism that gives high possibilities of maintaining security and privacy are required [48].

A main future extension is a study of how the proposed methodology is improved when additional information is added to it, both from the network and from the music education content. This would apply to both of these sources of data. In addition to this, we intend to study the impact that the amount of the data has on the algorithm's scalability and evaluate how well our method performs in additional private databases. It would be interesting also to investigate new ways of encoding the available information with crypto-tensioners to integrate this further information into the technique that has been proposed on a methodological level.

## Data Availability

The data used in this study are available from the author upon request.
